# Exposure of U.S. Children to Residential Dust Lead, 1999–2004: I. Housing and Demographic Factors

**DOI:** 10.1289/ehp.11917

**Published:** 2008-11-14

**Authors:** Joanna M. Gaitens, Sherry L. Dixon, David E. Jacobs, Jyothi Nagaraja, Warren Strauss, Jonathan W. Wilson, Peter J. Ashley

**Affiliations:** 1 Healthy Housing Solutions, Inc., Columbia, Maryland, USA;; 2 National Center for Healthy Housing, Columbia, Maryland, USA;; 3 Battelle Memorial Institute, Columbus, Ohio, USA;; 4 U.S. Department of Housing and Urban Development, Washington, DC, USA

**Keywords:** dust lead, housing, lead, National Health and Nutrition Examination Survey, NHANES

## Abstract

**Background:**

Lead-contaminated house dust is a major source of lead exposure for children in the United States. In 1999–2004, the National Health and Nutrition Examination Survey (NHANES) collected dust lead (PbD) loading samples from the homes of children 12–60 months of age.

**Objectives:**

In this study we aimed to compare national PbD levels with existing health-based standards and to identify housing and demographic factors associated with floor and windowsill PbD.

**Methods:**

We used NHANES PbD data (*n* = 2,065 from floors and *n* = 1,618 from windowsills) and covariates to construct linear and logistic regression models.

**Results:**

The population-weighted geometric mean floor and windowsill PbD were 0.5 μg/ft^2^ [geometric standard error (GSE) = 1.0] and 7.6 μg/ft^2^ (GSE = 1.0), respectively. Only 0.16% of the floors and 4.0% of the sills had PbD at or above current federal standards of 40 and 250 μg/ft^2^, respectively. Income, race/ethnicity, floor surface/condition, windowsill PbD, year of construction, recent renovation, smoking, and survey year were significant predictors of floor PbD [the proportion of variability in the dependent variable accounted for by the model (*R*^2^) = 35%]. A similar set of predictors plus the presence of large areas of exterior deteriorated paint in pre-1950 homes and the presence of interior deteriorated paint explained 20% of the variability in sill PbD. A companion article [Dixon et al. Environ Health Perspect 117:468–474 (2009)] describes the relationship between children’s blood lead and PbD.

**Conclusion:**

Most houses with children have PbD levels that comply with federal standards but may put children at risk. Factors associated with PbD in our population-based models are primarily the same as factors identified in smaller at-risk cohorts. PbD on floors and windowsills should be kept as low as possible to protect children.

The U.S. Centers for Disease Control and Prevention (CDC) estimates that 310,000 children between 1 and 6 years of age in the United States have blood lead (PbB) levels > 10 micrograms per deciliter (CDC 2005). The health effects associated with PbB levels at or above this level of concern have been well documented, including learning and behavioral problems (National Research Council 1993). Evidence suggests that children with PbB < 10 μg/dL also experience notable adverse effects and that no safe level of lead exposure exists (Canfield et al. 2003; CDC 1991; Lanphear et al. 2000, 2005b; Schwartz 1994). In this study we identified factors associated with childhood residential dust lead (PbD) exposure.

Lead exposure can occur through a variety of sources, including air, bare soil, home remedies, drinking water, toy jewelry, and others (Levin et al. 2008). However, the major pathway of exposure for children is from deteriorated lead-based paint and lead-contaminated dust in the home that is ingested during normal hand-to-mouth behavior (CDC 2007; Lanphear et al. 1998). The importance of PbD from lead paint was recognized very early (Gibson 1904), and work was done subsequently in an attempt to quantify its exposure contribution (Sayre et al. 1974).

Although lead-based paint was banned from residential use in 1978, approximately 38 million older housing units in the United States still contained lead-based paint, and an estimated 24 million housing units contain significant lead hazards as of 2000 (Jacobs et al. 2002). Although intact paint does not generally result in significant immediate exposure, all paint eventually deteriorates; lead-based paint that is chipping, peeling, or flaking or otherwise separating from its substrate presents a hazard. In addition, lead-contaminated settled dust, which is often found in houses with deteriorated lead-based paint, is a significant lead hazard (Lanphear et al. 1998). PbD can also be generated from the friction and impact of lead-painted surfaces (Dixon et al. 2007) and during housing renovation and repair projects where lead-based paint is present and proper precautions are not in place (Lanphear et al. 2005a; Reissman et al. 2002). The use of leaded gasoline, which peaked in the early 1970s, has also contaminated soil around the home (Mielke 1999). Many studies, employing a variety of research designs, have demonstrated that soil-lead concentrations are a significant contributor to PbD and children’s PbB (Bornschein et al. 1987; Clark et al. 2004; Dixon et al. 2007; Lanphear et al. 1998). Numerous cross-sectional (Lanphear et al. 1998) and longitudinal studies [U.S. Department of Housing and Urban Development (HUD) 2004] have firmly established the correlation of settled PbD and children’s PbB. In an effort to protect young children from adverse effects of lead, current federal health-based hazard standards indicate that floor and window PbD should not exceed 40 μg/ft^2^ and 250 μg/ft^2^, respectively [U.S. Environmental Protection Agency (EPA) 2001].

Through an interagency agreement with the CDC, the U.S. HUD Office of Healthy Homes and Lead Hazard Control sponsored the collection of PbD wipe samples and housing-related data through the National Health and Nutrition Examination Survey (NHANES) from 1999 through 2004, marking the first time that NHANES has collected both health and housing environmental data. Using these national survey data, we investigated PbD in homes to explore the feasibility of lowering PbD standards. Here we present the demographic and housing characteristics associated with floor and windowsill PbD. We used linear regression modeling to predict natural log-transformed floor and windowsill PbD and logistic regression modeling to predict the log odds of PbD at various levels. A companion article in this issue (Dixon et el. 2009) presents the analysis of NHANES data with respect to childhood PbB levels. Together these data identify the important risk factors and the relationship between PbD and children’s PbB in the United States in recent years.

## Methods

### Study population

We examined three waves of NHANES (1999–2000–2001–2002–2003–2004) data for children 12 to 60 months of age with measured PbB. Included in this population were homes of 2,155 children, of which 2,065 had floor PbD data and 1,618 had windowsill PbD data. NHANES is a nationally representative cross-sectional household survey that uses a complex, stratified, multistage probability sampling design to track the health of the noninstitutionalized civilian U.S. population. Details of the NHANES protocol, survey and analytical procedures, and handling of samples are available elsewhere [National Center for Health Statistics (NCHS) 2006a, 2006b, 2006c].

### Demographic and housing characteristics

NHANES interviewers collected demographic and housing information through a structured household interview. Characteristics included child’s race/ethnicity, household and family income, type of home (e.g., mobile home or trailer, one-family house detached, one-family house attached to one or more houses, apartment, or “other”), number of apartment units in building, year of construction, number of years the family had lived in the home, ownership status, and smoking in the home. Parents of participants reported the race/ethnicity of their child based on lists that included an open response. We used a composite race/ethnicity variable for this analysis: non-Hispanic white, non-Hispanic black, Hispanic (composed mostly of Mexican American because of oversampling), or other race or ethnicity. The poverty-to-income ratio (PIR) is the ratio of income to the family’s appropriate poverty threshold (Office of Management and Budget 1978). PIR values < 1.00 are below the poverty threshold, whereas PIR values of ≥ 1.00 indicate income above the poverty level. Variables on smoking behavior included the presence of smoking in the home, number of smokers, and the number of cigarettes smoked in the home per day. An adult member of the household reported the presence of peeling, chipping, or flaking paint (i.e., deteriorated) inside and outside the home. The household member categorized the paint condition inside the house as follows: no deteriorated paint, deteriorated paint but no large areas, large areas of deteriorated paint in one room, or large areas of deteriorated paint in more than one room. Similarly, the household member classified the exterior paint condition as no deteriorated paint, deteriorated paint but no large areas, or large areas of deteriorated paint. Large areas inside the home were defined as areas larger than one sheet of a newspaper and large areas on the outside of the home as areas larger than a door.

The household member also reported whether the home had been repainted, whether they had scraped old paint or whether there had been renovations of windows, cabinets, and/or walls in the preceding 12 months.

### PbD measurements

Interviewers collected separate single-surface floor and windowsill PbD samples from the room where the family member reported that children spent most of their time while awake, typically the living room or play room. Floor PbD wipe samples were collected from a measured 1 ft^2^ area in 2,065 homes using a standard procedure for moist wipes (ASTM E-1728-03; ASTM International 2003). Floor PbD was measured using graphite furnace atomic absorption spectroscopy and reported in micrograms per square foot. Windowsill PbD in 1,618 homes was also reported as micrograms per square foot using information on the length and width dimensions of the windowsill wiped area. The laboratory detection limits (DLs) for the moist wipe samples were 0.16 μg for floors and 2 μg for windowsills. Blank samples were collected in 10% of the sampled homes. Robust laboratory quality control procedures were followed (NCHS 2006d, 2006e, 2006f).

Floor and sill PbD values that were below the DLs were assigned the value of 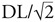 in the NHANES dust analysis data set. Forty-four percent of the sill loadings and 12.5% of the floor loadings were below the DLs. Although sill loadings are generally higher than floor PbD, the surface area of windowsills is typically less than the 1 ft^2^ sampled on floors, resulting in more sill loadings below the DL. The effects of the high percentage of sill loadings below the DL in the linear model are limited because the surface areas of sills sampled varied and consequently the loadings varied.

We categorized the floor’s surface and condition as uncarpeted smooth and cleanable, uncarpeted not smooth or not cleanable, low-pile carpet, or high-pile carpet. Windowsill conditions were characterized as either smooth and cleanable or not. The modeling presented here also examined room cleanliness, presence of clutter, and room location as reported by the individual collecting the wipe sample.

### Statistical methods

We analyzed data using SAS (SAS System for Windows, version 9.1.3; SAS Institute Inc., Cary, NC) and SUDAAN software (version 9.0.0; RTI International, Research Triangle Park, NC).

We used linear regression models to predict natural log-transformed PbD and logistic regression models to predict the probability that a home’s PbD exceeds various thresholds (10 μg lead/ft^2^ for floors and 100 and 250 μg/ft^2^ for windowsills). Models for 10 μg/ft^2^ for floors and 100 μg/ft^2^ for windowsills were selected because analyses in the companion article indicate that 95% of children 1–5 years of age would have PbB ≤ 10 μg/dL at these dust lead levels. Because only three homes in the data set had floor PbD exceeding the federal hazard standard of 40 μg/ft^2^, we could not use logistic regression modeling to predict the probability that floor PbD exceeded the standard. The models adjusted the parameter estimates for the clustering and unequal survey weights within NHANES. The modeling employed Taylor series expansion theory without degrees of freedom adjustments. Backward elimination of insignificant independent variables (*p* > 0.10) was followed by additional steps to allow addition and/or removal of variables. For certain variables, there was a high percentage of missing values (e.g., 28% of the study sample did not have year of construction documented). We fit an intercept term for the study participants who had a missing value so that we could include these homes in the analysis. For categorical variables, we reported the *p-*value for the test for a significant difference in the dependent variable between the category of interest and the comparison category, where the comparison category is the category with parameter estimate of zero. The overall *p-*value is the type 3 *F*-test that captures the overall statistical significance of each variable included in the model. For categorical variables with missing values, the missing level was not included in this hypothesis test.

Because the surface area and concentration of lead paint is higher in pre-1978 and particularly pre-1950 housing (Jacobs et al. 2002), our models allowed the effects of paint deterioration, renovation, repainting, and paint scraping in the preceding 12 months to be modified by the year of construction. This allowed paint deterioration, renovation, repainting, and paint scraping to have effects on PbD only in homes constructed before 1978 or before 1950.

Model diagnosis is complex for the analysis of data from a clustered multiframe survey with unequal weights, such as NHANES. Thus for the linear models, residual analysis to assess the validity of the assumption of normality of the error was based on models with the same predictors but ignoring the clustering and survey weights.

For the logistic model, we used analysis of deviance (McCullagh and Nelder 1989) to assess the goodness of fit of the model. Although this measure accounts for the survey weights, it does not account for the effects of clustering.

## Results

### Characteristics of the study population

Tables 1 and 2 provide descriptive statistics for the continuous and categoric variables that were significant in predicting floor and windowsill PbD in the linear and logistic regression models. The geometric means (GMs) for floor and windowsill PbD were 0.52 μg/ft^2^ and 7.64 μg/ft^2^, respectively. Only 0.16% of the weighted floor dust samples and 4.00% of the windowsill dust samples were at or above the current federal hazard standards. Most floor and sill samples (84.3%) were collected in family rooms, living rooms, or dens. Nearly 10% of the samples were collected in bedrooms, 1.7% from kitchens, 1.4% from dining rooms, and 3.10% from another room. Floor dust samples were primarily from carpeted areas (80.12%). Only 1% of floor dust samples and 10% of windowsill samples were from nonsmooth or noncleanable hard surfaces. However, uncarpeted floor surfaces that were not smooth or not cleanable had higher GM PbD than did smooth and cleanable surfaces (1.7 μg/ft^2^ vs. 1.0 μg/ft^2^). Floor PbD from smooth and cleanable surfaces were more than double PbD on low-pile and high-pile carpeted surfaces (1.0 μg/ft^2^ vs. 0.46 μg/ft^2^ and 0.35 μg/ft^2^, respectively). Approximately 21% of homes had smoking occurring inside the home, 22% of homes had reported areas of deteriorated paint inside the home, 52% of homes were built before 1978, and 4% of homes were constructed before 1950 and had recent renovations. Only 1.7% of homes reported exterior deteriorated paint, possibly because of the large surface area required for this category.

### Floor PbD linear model

Table 3 provides the parameter estimates and associated SEs for the linear model that predicts natural log-transformed floor PbD.

Floor PbD was not significantly different between housing built from 1978 to 1989 and that from 1990 to present, although the difference between pre- and post-1978 was significant (*p* < 0.001). The difference in PbD by race/ethnicity was also significant, with non-Hispanic blacks having significantly higher levels than non-Hispanic whites (*p* < 0.001). PbD in Hispanic homes was not significantly different than non-Hispanic white homes (*p* = 0.864). A higher PIR was significantly associated with lower PbD (*p* = 0.021). Higher windowsill PbD was significantly associated with higher floor PbD (*p* < 0.001). Floor PbD in the 1999–2000 NHANES was significantly higher than floor PbD in the 2001–2002 or 2003–2004 waves (both *p* < 0.001). The presence of a smoker in the home was associated with significantly higher floor PbD (*p* < 0.006), as was window, cabinet, or wall renovation in pre-1950 homes (*p* = 0.056).

Although floor PbD on uncarpeted nonsmooth or noncleanable floors was higher than on smooth and cleanable floors, this difference was not statistically significant (*p* = 0.170), perhaps because only 25 homes had uncarpeted floors that were not smooth and cleanable.

This multivariate statistical model explains 35% of the variability in floor PbD of a home. If a variable was significant in either the linear or logistic model but not the other model, the cells for that variable in the model that did not contain the variable in Table 3 contain a dash (—).

### Floor PbD logistic model

Only 1.92% of homes had floor PbD ≥ 10 μg/ft^2^ (Table 2). The parameter estimates for the log-odds that floor PbD is ≥ 10 μg/ft^2^ are shown in Table 3. Our results indicate that PbD from high-pile or low-pile carpet is much less likely to exceed 10 μg/ft^2^ than PbD from smooth and cleanable floors (*p* < 0.001 and 0.024, respectively). Floor PbD in homes of non-Hispanic blacks was higher than in homes of non-Hispanic whites (*p* = 0.088). Year of construction, type of home or apartment, and sill PbD were also significant predictors of floor PbD ≥ 10 μg/ft^2^. Several variables, including PIR, smoking in the home, renovation, and survey year, were significant in the linear regression model but were not significant in the logistic model.

### Windowsill PbD linear model

Table 4 provides the parameter estimates and associated SEs for the linear model that predicts natural log-transformed windowsill PbD.

Homes built after 1950 had lower windowsill PbD compared with those built before 1950 (*p* < 0.001). The presence of deteriorated interior paint was associated with higher sill PbD (*p* = 0.028). Pre-1950 homes that had large areas of deteriorated paint on the outside of the home also had higher sill PbD than other homes (*p* = 0.076). Homes of non-Hispanic blacks had significantly higher sill PbD than homes of non-Hispanic whites (*p* < 0.001). Sill PbD in homes of Hispanics were not significantly different from that in homes of non-Hispanic whites (*p* = 0.298). Smoking inside the home was also positively associated with sill PbD (*p* = 0.001). Samples taken from surfaces that were not smooth or not cleanable had significantly higher PbD compared with samples taken from smooth and cleanable surfaces (*p* = 0.009), as was the case for floors. Similar to the linear floor PbD model, the year of the survey was statistically significant. The sill PbD in 1999–2000 was higher than in the 2001–2002 or 2003–2004 waves (*p* = 0.006).

The multivariate statistical model explains 20% of the variability in natural-log transformed windowsill PbD.

### Windowsill PbD logistic regression models

Only 8.90% of the homes had windowsill PbD ≥ 100 μg/ft^2^, and 4.00% of homes yielded windowsill PbD ≥ 250 μg/ft^2^ (Table 2). The parameter estimates for the log-odds that a windowsill PbD is ≥ 100 μg/ft^2^ and ≥ 250 μg/ft^2^ are shown in Table 5.

In both logistic models, smoking in the home and year of construction were statistically significant predictors. If someone smoked inside the home, the odds that the sill PbD was ≥ 100 or ≥ 250 μg/ft^2^ were nearly 90% higher than if no one smoked inside the home.

Interestingly, in the logistic model for PbD ≥ 100 μg/ft^2^, the odds that sill PbD was ≥ 100 μg/ft^2^ for homes with large areas of exterior deteriorated paint was about three times higher than for homes with no exterior deteriorated paint. In the logistic model for PbD ≥ 250 μg/ft^2^, if the interior paint deterioration was large in two or more rooms, the odds that sill PbD was ≥ 250 μg/ft^2^ were about three times higher than if there was no interior paint deterioration.

Most variables that achieved statistical significance did so in more than one of the three sill PbD models, making these findings robust. For example, smoking and year of building construction were significant in all three models. Paint scraping in the preceding 12 months and the years lived in the home were only significant in the logistic model for PbD ≥ 100 μg/ft^2^. Window surface condition was only significant in the linear model.

## Discussion

Consistent with other national data (Jacobs et al. 2002), we confirm that the year of construction is a strong predictor of PbD and that post-1978 housing has significantly lower PbD than older housing. Housing units built after 1950 have significantly lower floor PbD than older housing. Furthermore, floor PbD in houses built between 1940 and 1949 are not significantly different than in the pre-1940 houses. This is consistent with the concentration of lead in paint. Before 1940, this concentration typically ranged from 10 to 50% (Rabin 1989; U.S. HUD 1995). However, in 1955, a voluntary paint industry standard limited the concentration to 1%, although the degree of compliance with this standard is unknown (American National Standards Institute 1955). In 1978, the Consumer Product Safety Commission and Congress banned the use of lead paint for residential use, limiting lead in new house paint to 0.06% by weight (Consumer Products Safety Commission 1977).

Pre-1950 homes with window, cabinet, or wall renovation within the preceding 12 months had higher floor PbD than other homes. Renovation activities completed without using lead-safe work practices can generate significant amounts of PbD (Jacobs et al. 2003). The U.S. EPA recently promulgated a final regulation in an attempt to control exposures from renovation (U.S. EPA 2008). Replacing windows in a lead-safe manner can help control PbD and may have large economic benefits (Nevin et al. 2008). Compared with other housing components, windows are known to have the highest levels of lead-based paint and lead-contaminated dust (Jacobs et al. 2002).

We found that dust wipe samples taken from homes with chipping, peeling, or flaking (i.e., deteriorated) paint had higher windowsill PbD than homes without deteriorated paint, which is consistent with other research (U.S. HUD 2004; Wilson et al. 2006). Although we expected to see similar findings for floors, we did not. This could be attributable to other factors included in the model. For example, PIR and recent renovation were significant in the floor model, but not in the windowsill model, which could have masked the effect of deteriorated paint. In addition, it is more likely that floors are more regularly cleaned than windowsills.

The homes of non-Hispanic blacks had significantly higher PbD than the homes of non-Hispanic whites, even after controlling for other factors. Many prior studies have shown that African-American children are at higher risk compared with white children (U.S. CDC 2005). For example, similar to our findings, other studies (Lanphear et al. 1996, 2002) found that African-American children were exposed to higher PbD loadings and worse housing conditions than white children.

Previously published studies suggest that rental units were more likely to have lead-based paint hazards than owner-occupied housing (Jacobs et al. 2002; Lanphear et al. 2005a). Although we found that homeownership status was a significant predictor of floor PbD in bivariate analysis, after controlling for other factors such as PIR, renovation, and the presence of deteriorating paint, it was not significant in the final models. Because low-income families are more likely to rent, the fact that home ownership was not significant in the model (but poverty was) is not surprising. Moreover, the type of home had a significant association with PbD in bivariate analysis, but not in the final models, again probably because of the confounding influence of poverty.

A prior study found a relationship between exposure to environmental tobacco smoke exposure and the PbB levels of young children who were included in the NHANES III (1988–1994) (Mannino et al. 2003). Because lead is a component of tobacco smoke, such a relationship between PbB levels and tobacco smoke exposure is not surprising. That smoking in the home was a significant predictor of floor and windowsill PbD, even when controlling other factors, suggests that lead in secondhand smoke is a significant contributor to lead on interior surfaces, at least at the relatively low surface loadings documented in this study.

We found that the surface condition from which the PbD samples were taken significantly influences the reported PbD. Other studies have found that uncarpeted smooth and cleanable surfaces have significantly lower PbD after cleaning compared with rough uncarpeted surfaces (Dixon et al. 1999; Ettinger et al. 2002). Although our results indicated that floor PbD on uncarpeted non-smooth or noncleanable surfaces was not significantly higher than floor PbD on smooth and cleanable surfaces, the trend was in the expected direction. It is possible that our findings were not statistically significant because of the small number of dust samples taken from nonsmooth or noncleanable floor surfaces in this data set.

The presence of carpeting also influenced reported PbD. We found that PbD on carpeted floors was significantly lower than lead loadings on hard-surfaced floors. This observed difference in lead loading by flooring type is likely attributable to the fact that wipe sampling was the methodology used in this study. Wipe sampling captures only dust adhering to the tops of the carpet fibers, whereas most of the dust in the carpet matrix is located deeper in the pile. A study that reported much higher PbD in vacuum samples from carpet compared with adjacent wipe samples (Bai et al. 2003) supports this idea. The significance of this observation with respect to children’s lead exposure is not well understood. Our findings do not clarify whether or not carpet contributes to higher or lower exposures.

The NHANES survey year was significantly associated with PbD. Although PbD should decline with time as the ratio of post-1978 to pre-1978 homes increases, the magnitude of the decline (14%) for the floor PbD between the first and second waves was much larger than expected. Similarly, the magnitude of the decline (43%) between the first and second waves for windowsill PbD is unlikely to be explained only by temporal changes in national PbD. Although many housing characteristics were included in the model, other significant factors that we were unable to control for may exist (e.g., different waves of NHANES sampling may occur in different geographic regions). Thus, the year of survey variable may reflect geographic differences in the three study wave locations that could account for the observed trend in floor PbD.

We used data from a large national survey that combined housing and environmental data, which is a strength of our study. However, the housing data included in this study may not be representative of the national housing stock. The sampling and weighting methodology used in NHANES is population based, not geography based; thus, the survey includes a nationally representative sample of the U.S. population but not a representative sample of U.S. housing. Integrated health and housing surveys that are representative of both the population and the housing stock are needed in the United States; such surveys were completed recently in eight European cities (Bonnefoy et al. 2003). Finally, a limitation of the NHANES PbD data is that they are based on a single floor and a single windowsill PbD measurement in a given home. The HUD *Guidelines for the Evaluation and Control of Lead-Based Paint Hazard in Housing* recommend that six to eight floor and sill samples be taken to help reduce spatial variability, which could not be assessed here (U.S. HUD 1995). Despite these potential limitations, the study results presented are largely consistent with earlier findings.

Levels of PbD on floors and windowsills should be kept as low as possible to protect children from lead exposure. The current standards for floor and windowsill PbD were set in 1999–2001 to protect 95% of children from developing a PbB level > 15 μg/dL [the environmental intervention level established by the CDC (2005)], in light of feasibility and measurement limitations. These findings show that in most children’s homes, the average level of PbD is well below the current standards, making it feasible to lower the current standards and thus afford more protection for children.

## Figures and Tables

**Table 1:** Descriptive statistics for continuous housing and demographic variables, NHANES 1999–2004.

			Weighted	
Variable	Levels	No. of units	GM (GSE)	AM (SE)
Floor PbD by floor surface/condition	Missing	90	—	*—*
	Smooth and cleanable	453	0.99 (1.11)	3.16 (0.56)
	Not smooth and cleanable	25	1.70 (1.47)	4.92 (2.11)
	Carpeted, low pile	1,381	0.46 (1.06)	0.91 (0.11)
	Carpeted, high pile	206	0.35 (1.10)	0.62 (0.08)
	All nonmissing	2,065	0.52 (1.05)	1.34 (0.14)
Windowsill lead loading by window surface condition	Missing	537	—	
	All nonmissing	1,618	7.64 (1.07)	57.79 (9.42)
	Smooth and cleanable	1,453	6.98 (1.07)	47.57 (5.30)
	Not smooth and cleanable	165	16.9 (1.24)	146.6 (69.35)
PIR^a^	Missing	136	—	—
	Nonmissing	2,019	—	2.07 (0.05)
Years lived in home^a^	Missing	23	—	—
	Nonmissing	2,132	—	2.64 (0.04)

Abbreviations: AM, arithmetic mean; GSE, geometric standard error.

a GM and GSE are undefined because of zero values.

**Table 2:** Descriptive statistics for categorical housing and demographic variables, NHANES 1999–2004.

			Weighted percent	
Variable	Levels	No. of units	Missing included	Missing excluded
Floor PbD loading ≥ 40 μg/ft^2^	Missing	90	3.68	—
	No	2,062	96.17	99.84
	Yes	3	0.15	0.16

Floor PbD loading ≥ 10 μg/ft^2^	Missing	90	3.68	—
	No	2,006	94.40	98.00
	Yes	59	1.93	2.00

Floor surface/condition	Missing	90	3.68	—
	Smooth and cleanable	453	18.18	18.87
	Not smooth and cleanable	25	0.96	1.00
	Carpeted, low pile	1,381	66.08	68.60
	Carpeted, high pile	206	11.1	11.52

Windowsill PbD loading > 100 μg/ft^2^	Missing	537	24.17	—
	No	1,465	69.07	96.00
	Yes	153	6.75	4.00

Windowsill PbD loading ≥ 250 μg/ft^2^	Missing	537	24.17	—
	No	1,538	72.8	91.10
	Yes	80	3.03	8.90

Window surface condition	Missing	537	24.17	—
	Smooth and cleanable	1,453	68.00	89.67
	Not smooth and cleanable	165	7.83	10.33

Room dust sampled	Missing	90	3.68	—
	Living room, family room, or den	1,700	81.20	84.30
	Dining room	29	1.31	1.36
	Kitchen	33	1.66	1.73
	Bedroom	250	9.17	9.52
	Another room	53	2.98	3.10

Year of construction	Missing	840	28.1	—
	1990–present	287	19.61	27.28
	1978–1989	265	14.84	20.64
	1960–1977	304	14.35	19.96
	1950–1959	168	7.43	10.34
	1940–1949	82	4.27	5.94
	Before 1940	209	11.39	15.84

Window, cabinet, or wall renovation in pre-1950 home	Missing	174	5.97	—
	Yes	70	3.98	4.23
	No	1,911	90.05	95.77

Anyone smoke inside the home	Missing	23	1.50	—
	Yes	430	20.78	21.09
	No	1,702	77.73	78.91

Year of survey	1999–2000	624	30.23	30.23
	2001–2002	765	34.08	34.08
	2003–2004	766	35.69	35.69

Extent of peeling, flaking, or chipping paint outside	Missing	392	15.88	—
	No deteriorated paint	1,376	64.57	76.75
	Deteriorated paint but no large areas	309	15.98	18.99
	Large areas of deteriorated paint	78	3.58	4.26

Presence of large area of deteriorated paint outside in pre-1950 home	Missing	283	10.42	—
	Yes	27	1.55	1.73
	No	1,845	88.03	98.27

Extent of paint deterioration inside	Missing	28	1.45	—
	No deteriorated paint	1,596	76.73	77.86
	Deteriorated paint but no large areas	439	18.75	19.03
	Large areas of deteriorated paint in one room	65	2.33	2.36
	Large areas of deteriorated paint in two or more rooms	27	0.74	0.75

Presence of paint deterioration inside	Missing	28	1.45	—
	Yes	531	21.82	22.14
	No	1,596	76.73	77.86

Paint scraped when home repainted in preceding 12 months	Missing	1,423	59.59	—
	Yes	197	10.63	26.44
	No	535	29.57	73.56

Race/ethnicity	Non-Hispanic white	618	57.09	57.09
	Non-Hispanic black	634	15.32	15.32
	Hispanic^a^	837	23.82	23.82
	Other	66	3.77	3.77

a Sixty-six percent of Hispanics are Mexican Americans.

**Table 3:** Model results for floor PbD.

	Linear model for log PbD (*R*^2^ = 35%)			Logistic model PbD ≥ 10 μg/ft^2^ (*R*^2^ = 7%^a^)		
Variables	Overall *p-*value	Estimate (SE)	*p-*Value	Overall *p-*value	Estimate (SE)	*p-*Value
Intercept	0.235	−0.239 (0.199)	0.235	< 0.001	−6.179 (0.834)	< 0.001
Floor surface/condition						
Smooth and cleanable	< 0.001	0.000		< 0.001	0.000	
Not smooth and cleanable		0.354 (0.254)	0.170		0.449 (0.740)	0.547
Carpeted, low pile		−0.634 (0.094)	< 0.001		−2.147 (0.401)	< 0.001
Carpeted, high pile		−0.868 (0.110)	< 0.001		−2.868 (1.229)	0.024
Log windowsill PbD loading						
Intercept for missing	< 0.001	0.409 (0.090)	< 0.001	< 0.001	0.000	
Slope		0.172 (0.027)	< 0.001		0.732 (0.106)	< 0.001
Race/ethnicity						
Non-Hispanic white	< 0.001	0.000	—	0.009	0.000	—
Non-Hispanic black		0.373 (0.086)	< 0.001		0.900 (0.516)	0.088
Hispanic		−0.015 (0.087)	0.864	—	—	—
Other^b^		−0.194 (0.129)	0.140	—	−0.492 (0.581)	0.402
PIR						
Intercept for missing	0.028	0.036 (0.120)	0.768	—	—	—
Slope		−0.047 (0.020)	0.021	—	—	—
Year of construction						
Intercept for missing	< 0.001	−0.136 (0.141)	0.338	< 0.040	−0.032 (0.665)	0.962
1990–present		−0.795 (0.128)	< 0.001		—	—
1978–1989		−0.714 (0.149)	< 0.001		—	—
1960–1977		−0.410 (0.137)	0.004		—	—
1950–1959		−0.366 (0.177)	0.044		0.872 (0.723)	0.234
1940–1949		−0.393 (0.242)	0.118		1.284 (1.015)	0.213
Before 1940		0.000	—		0.000	—
1960–present		—	—		−1.519 (0.775)	0.056
Anyone smoke inside the home						
Intercept for missing	0.006	−0.352 (0.344)	0.312	—	—	—
Yes		0.253 (0.087)	0.006		—	—
No		0.000			—	—
Window, cabinet, or wall renovation in a pre-1950 home						
Intercept for missing	0.056	−0.113 (0.116)	0.334	—	—	—
Yes		0.355 (0.181)	0.056		—	—
No	0.000				—	—
Year of survey						
1999–2000	< 0.001	0.429 (0.093)	< 0.001	—	—	—
2001–2002		0.067 (0.091)	0.470		—	—
2003–2004		0.000			—	—
Home-apartment type						
One-family house, detached		—	—	0.042	1.032 (0.550)	0.067
One-family house, attached		—	—		1.397 (0.739)	0.066
Apartment (1–9 units)		—	—		1.964 (0.604)	0.002
Apartment (≥ 10 units)		—	—		0.000	

a Estimated using Cox–Snell methodology.

b “Other” includes Hispanics for the logistic model.

**Table 4:** Linear model results for log windowsill PbD (*R*^2^ = 20%).

Variable	Levels	Overall *p-*value	Estimate (SE)	*p-*Value
Intercept		< 0.001	2.670 (0.190)	< 0.001
Race/ethnicity	Non-Hispanic white	0.001	0.000	—
	Non-Hispanic black		0.521 (0.114)	< 0.001
	Hispanic		0.145 (0.138)	0.298
	Other		−0.234 (0.241)	0.338
Year of construction	Intercept for missing	< 0.001	−0.777 (0.234)	0.002
	1990–present		−1.616 (0.249)	< 0.001
	1978–1989		−1.442 (0.216)	< 0.001
	1960–1977		−1.332 (0.221)	< 0.001
	1950– 1959		−1.072 (0.315)	0.001
	1940–1949		−0.715 (0.410)	0.088
	Before 1940		0.000	—
Window surface condition	Smooth and cleanable	0.001	0.000	—
	Not smooth and cleanable		0.759 (0.213)	0.001
Anyone smoke inside the home	Intercept for missing	0.001	0.664 (0.824)	0.425
	Yes		0.460 (0.130)	0.001
	No		0.000	
Presence of large area of deteriorated paint outside in pre-1950 home	Intercept for missing	0.076	−0.422 (0.200)	0.040
	Yes		0.992 (0.545)	0.076
	No		0.000	—
Presence of paint deterioration inside	Intercept for missing	0.028	−0.413 (0.649)	0.528
	Yes		0.361 (0.159)	0.028
	No		0.000	—
Year of survey	1999–2000	0.020	0.330 (0.144)	0.027
	2001–2002		−0.100 (0.114)	0.382
	2003–2004		0.000	—

**Table 5:** Logistic model results for windowsill PbD.^a^

		PbD ≥ 100 μg/ft^2^ (*R*^2^ = 7%^a^)			PbD ≥ 250 μg/ft^2^ (*R*^2^ = 4%^a^)		
Variable	Levels	Overall *p-*Value	Estimate (SE)	*p-*Value	Overall *p-*Value	Estimate (SE)	*p-*Value
Intercept		< 0.001	−1.289 (0.385)	0.002	< 0.001	−2.502 (0.352)	< 0.001
Race/ethnicity	Non-Hispanic white	—	—	—	0.002	0.000	—
	Non-Hispanic black	—	—	—		1.127 (0.297)	< 0.001
	Other^b^	—	—	—		0.049 (0.569)	0.932
Year of construction	Intercept for missing	0.005	−1.162 (0.354)	0.002	< 0.001	−1.161 (0.386)	0.004
	1990–present		−2.194 (0.805)	0.009		−3.201 (0.812)	< 0.001
	1978–1989		−1.852 (0.542)	0.001		−2.038 (0.895)	0.028
	1960–1977		−1.603 (0.365)	< 0.001		−1.705 (0.464)	0.001
	1950–1959		−1.045 (0.507)	0.045		−1.009 (0.624)	0.113
	1940–1949		−0.193 (0.521)	0.713		−0.121 (0.576)	0.834
	Before 1940		0.000	—		0.000	—
Anyone smoke inside the home	Intercept for missing	0.041	−0.336 (1.046)	0.749	0.059	10.472 (0.895)	< 0.001
	Yes		0.623 (0.296)	0.041		0.625 (0.323)	0.059
	No		0.000			0.000	—
Extent of paint deterioration inside	Missing	—	—	—	0.005	−10.017 (0.385)	< 0.001
	No deteriorated paint		—	—		0.000	—
	Deteriorated paint but no large areas		—	—		−0.044 (0.417)	0.916
	Large areas in one room		—	—		−1.402 (1.058)	0.192
	Large areas in two or more rooms		—	—		1.458 (0.669)	0.035
Extent of peeling, flaking, or chipping paint outside	Intercept for missing	0.005	−0.530 (0.456)	0.251	—	—	—
	No deteriorated paint		0.000	—		—	—
	Deteriorated paint but no large areas		−0.317 (0.364)	0.389		—	—
	Large areas of deteriorated paint		1.303 (0.586)	0.031		—	—
Paint scraped when home repainted in last 12 months	Intercept for missing	0.053	0.433 (0.293)	0.146	—	—	—
	Yes		0.899 (0.451)	0.053		—	—
	No		0.000	—		—	—
Years lived in the home	Intercept for missing	0.076	0.000	—	—	—	—
	Slope		−0.227 (0.124)	0.076		—	—
Year of survey	1999–2000	0.050	0.534 (0.229)	0.024	—	—	—
	2001–2002		−0.087 (0.306)	0.776	—	—	—
	2003–2004		0.000	—	—	—	—

a Estimated using Cox–Snell methodology.

b “Other” includes Hispanics.
